# The Shape of Water Stream Induces Differences in P300 and Alpha Oscillation

**DOI:** 10.3389/fnhum.2019.00460

**Published:** 2020-01-20

**Authors:** Noriaki Kanayama, Shumpei Mio, Ryohei Yaita, Takahiro Ohashi, Shigeto Yamawaki

**Affiliations:** ^1^Human Informatics Research Institute, National Institute of Advanced Industrial Science and Technology (AIST), Tsukuba, Japan; ^2^Center for Brain, Mind and KANSEI Sciences Research, Hiroshima University, Hiroshima, Japan; ^3^TOTO Limited, Research Institute, Chigasaki, Japan

**Keywords:** water, EEG, P300-event related potential, alpha oscillations, touch

## Abstract

Touching is a fundamental human behavior used to evaluate objects in the external world. Many previous studies have used tactile stimulation to conduct psychological and psychophysiological experiments. However, most of these studies used solid material, not water stream, as an experimental stimulus. To investigate water perception, or to easily control the temperature of an experimental stimulus, it is important to be able to control the water stimulus. In this study, we investigated the usability of water as an experimental stimulus for electroencephalography (EEG) experiments and report the basic EEG response to water stimulus. We developed a tactile stimulation device using a water stream to study EEG responses, with the ability to control the stimulus onset timing. As stimuli, we selected two types of water stream, normal and soft, based on a psychological experiment to confirm a difference of subjective feeling induced by these water streams. We conducted a typical oddball task using the two different water streams and recorded EEG waveforms from 64 electrodes while participants touched the water streams. We calculated P300 at the Pz electrode, alpha asymmetry at the frontal electrodes, and alpha suppression at the parietal area. As a result, we observed typical P300 differentiation based on the stimulus proportion (target 20% and standard 80%). We observed a weaker alpha suppression when participants touched the soft water stream compared to the normal shower. These results demonstrate the usability of water stream in psychophysiological studies and suggested that alpha suppression could be a candidate to evaluate comfort of water stream.

## Introduction

Recent technologies for modulation of tactile experiences demonstrated that, in the very near future, tactile perception might be freely created to induce a specific affective response, in the same way that is currently possible with visual and auditory perception. For example, the softness experience could be modulated with vibrotactile stimuli (Hayward, 2008; Visell and Okamoto, 2014). These stimuli could not only be induced with typical solid materials but possibly with ultrasonic mid-air stimulation (Hoshi et al., 2009; Long et al., 2014).

A water stream is an ethologically important stimulus for inducing tactile perception on the surface of the human body. We often touch water in ordinary life, such as when washing one’s face, hand washing, dishwashing, toothbrushing, bathing, and drinking. Some uses of water include cleaning one’s body; in this regard, touching water can induce a comforting feeling. To elucidate the neural mechanisms underscoring the pleasure of touching, water may be a useful stimulation.

Experimental stimuli should be shared across laboratories to enhance replicability of results obtained from different cognitive neuroscience studies. For this reason, many researchers developed homemade devices for precise control of the experience of touching solid materials (McGlone et al., 2012; Muñoz et al., 2014; Kanayama et al., 2019). However, this is not always possible, as materials to be touched in an experiment vary greatly (Sakamoto and Watanabe, 2017).

The shape of a water stream could be altered by changing the shape of a faucet. Water stimulation can easily be reproduced in any location using an identical faucet shape. The different shapes of water stream could have different impacts on the subjective feeling of affective evaluation, including tactile comfort and richness of the water stream. Comfortable touching experiences induced by water stream could be modeled by affective/Kansei engineering (Nagamachi, 1995) using subjective sense of comfort about the water stream. Based on subjective reports of comfort about various water streams, the shape of a water stream can be optimized. However, subjective reports could be varied and modified by spontaneous appraisal, which makes stable modeling based on subjective reports challenging. Psychophysiological measurements, as an objective index, can support the model of subjective feelings of comfort (Balters and Steinert, 2017). To this end, we explored electroencephalography (EEG) components as a reflection of tactile comfort during perception of touching a water stream. However, EEG components are also not always more reliable than subjective reports, as they can vary depending on mental health, age, and current arousal state. Measurement of implicit and automatically activated evaluation of affective experience could potentially provide further insight not obtained from explicit reflective and subjective verbal reports.

To relate subjective comfort from touching a water stream, we used two typical EEG components of alpha band oscillation. Alpha oscillations are related to emotional responses, as the generation of alpha oscillations stems from regional cerebral blood flow in various cortical areas including emotion-related areas, such as the amygdala, basal prefrontal cortex, and insula (Sadato et al., 1998).

The first component we focused on in this study was alpha suppression, which is typically observed after stimulus presentation. Previous EEG studies using tactile stimuli have repeatedly showed alpha/beta band suppression after stimulation (van Ede et al., 2011; Singh et al., 2014). Singh et al. (2014) reported that beta band mu-suppression and beta-band oscillation showed a relationship with tactile caressing and subjective ratings of pleasantness. Bauer et al. (2006) reported that parieto-occipital alpha/beta suppression was modulated by spatial attention. This component over the parieto-occipital distribution has been observed in response to audiovisual stimulation, suggesting that this component is generated by cognitive processes independent of sensory modality (Schelenz et al., 2013). Some studies demonstrated that this component was more strongly suppressed when participants perceived a negative emotional stimulus compared to when perceiving a neutral stimulus (Jessen and Kotz, 2011; Swingle, 2013). Kostyunina and Kulikov (1996) reported that a decrease in alpha power was related to negative emotional states of fear and sorrow. These findings spurred us to measure participants’ emotional states using alpha suppression. We hypothesized that a softer stream would elicit weaker alpha suppression compared to a normal shower stream.

The second component we focused on was alpha asymmetry. This EEG component has repeatedly been observed when viewing emotional movie clips (Killeen and Teti, 2012; Lopez-Duran et al., 2012; Meyer et al., 2014; Zhao et al., 2018). Evidence that frontal asymmetry is modulated by tactile comfort is scarce; furthermore, physical distress during sleep can have an impact on this component (Flo et al., 2011). Thus, we aimed to investigate whether touching a soft water stream would induce alpha asymmetry. We hypothesized that a softer stream would elicit greater alpha asymmetry compared to a normal shower stream.

The difficulty of controlling a water stream has typically precluded the use of water as a tactile stimulus, especially in neuroscience and psychophysiology research. To our knowledge, no psychophysiological study using a water stream has been undertaken to date. Here, we examined using a water stream as stimulus in a typical oddball paradigm and confirmed that a water stream can induce a typical P300 component. By this investigation, we demonstrate the usability of water in psychophysiological studies, alongside solid and visual stimuli.

## Materials and Methods

### Pilot Study for Stimulus Selection

For this study, it was necessary to be able to compare whether or not pleasant touch was elicited in response to different shapes of water streams. To select stimuli, we conducted a pilot experiment on subjective evaluation of water streams. Fifteen people (five females, 10 males) participated in the pilot experiment. The mean age of participants was 31.67 years (*SD* = 5.95; range = 25–46). Twelve of these people also participated in the main experiment with EEG measurements. The pilot experiment was performed more than a year before the EEG experiment.

Participants evaluated five types of water streams (Table 1) in terms of “richness” and “comfort” after touching the water streams freely. For “richness,” participants rated whether they have “touched a high-quality thing:” 1 for very low quality, 9 for very high quality, and 5 for average quality. For “comfort,” participants rated whether they “felt comfort” when touching the water stream: 1 for high discomfort, 9 for high comfort, and 5 for neither. The difference between “comfort” and “richness” was the evaluation target. Comfort was the evaluation of the participant’s state, which is induced by the touch of water. Richness was the evaluation of the water stream itself. We could assume, for example, a case in which a participant feels richness on touching the water stream but does not feel comfort. To make the evaluation easier, participants were instructed to touch a flow with an aerator before the experiment started, an item not included in the stimulus list. Participants were instructed to consider this as the baseline and evaluate all other stimuli relative to the baseline score of 5. During the experiment, all water streams were hidden by a wall to exclude visual effects from the stimuli. The amount of water flowing was identical for each water stream, which was monitored by a flowmeter (sensor: FD-MH10A, display: FD-MA1A, KEYENCE, Osaka, Japan) and controlled within a range of ±0.2 L/min. The temperature of the water was kept within 23–25°C. Other physical properties of the two stimuli are listed in Table 1.

**Table 1:** Physical properties of water streams used in the pilot experiment.

Shape of flow	Comfort mean (SD)	Richness mean (SD)	Amount of water flowing (L/min)	Hole diameter (mm)	Hole numbers	Aperture area (mm^2^)
Normal shower	1.73 (0.88)	1.60 (0.91)	3.0	0.6	18	5.09
Soft flow	7.47 (1.36)	7.67 (0.82)	3.0	1.5	19	33.56
Laminar	7.00 (1.07)	6.80 (1.15)	3.0	12.5	1	122.66
Modified Soft flow	7.13 (1.49)	7.60 (1.18)	3.0	1.5	18	31.81
Numerous hole shower	5.87 (1.46)	1.73 (0.88)	3.0	-	82	43.36
Flow with aerator	5.00 (0.00)	5.00 (0.00)	3.0	11.9	1	111.16

To confirm pairs of water streams with different subjective feelings, we statistically analyzed comfort and richness scores for all pairs of stimuli. We observed significant differences in comfort scores between the pairs of normal shower vs. soft flow (*t*_(14)_ = −14.94, *p* < 0.001, Cohen’s *d* = −3.86), normal shower vs. laminar (*t*_(14)_ = −14.19, *p* < 0.001, Cohen’s *d* = −3.66), normal shower vs. modified soft flow (*t*_(14)_ = −11.34, *p* < 0.001, Cohen’s *d* = −2.93), Normal shower vs. numerous hole shower (*t*_(14)_ = −9.75, *p* < 0.001, Cohen’s *d* = −2.52) and soft flow vs. numerous hole shower (*t*_(14)_ = −3.36, *p* < 0.05, Cohen’s *d* = −0.87). We also observed significant differences in richness scores between pairs of normal shower vs. soft flow (*t*_(14)_ = −25.25, *p* < 0.001, Cohen’s *d* = −6.52), normal shower vs. laminar (*t*_(14)_ = −14.38, *p* < 0.001, Cohen’s *d* = −3.71), normal shower vs. modified soft flow (*t*_(14)_ = −18.33, *p* < 0.001, Cohen’s *d* = −4.73), normal shower vs. numerous hole shower (*t*_(14)_ = −10.81, *p* < 0.001, Cohen’s *d* = −2.79), soft flow vs. numerous hole shower (*t*_(14)_ = −7.74, *p* < 0.001, Cohen’s *d* = −2.00), and modified soft flow vs. numerous hole shower (*t*_(14)_ = −6.17, *p* < 0.01, Cohen’s *d* = −1.59). All *p*-values were corrected by Bonferroni method. Based on these results, we selected normal shower and soft flow as the paired stimuli for the EEG experiment.

### Participants

Thirty healthy individuals (15 females, 15 males) participated in the EEG experiment. Mean age of participants was 31.28 years (*SD* = 5.60; range = 24–46). All participants were employees of TOTO Limited. None of the participants were informed of the experimental aims before participation. Based on the ethical guideline of TOTO Limited, all participants provided oral informed consent before participation.

### Materials

#### Stimulation Device

**Figure 1:**
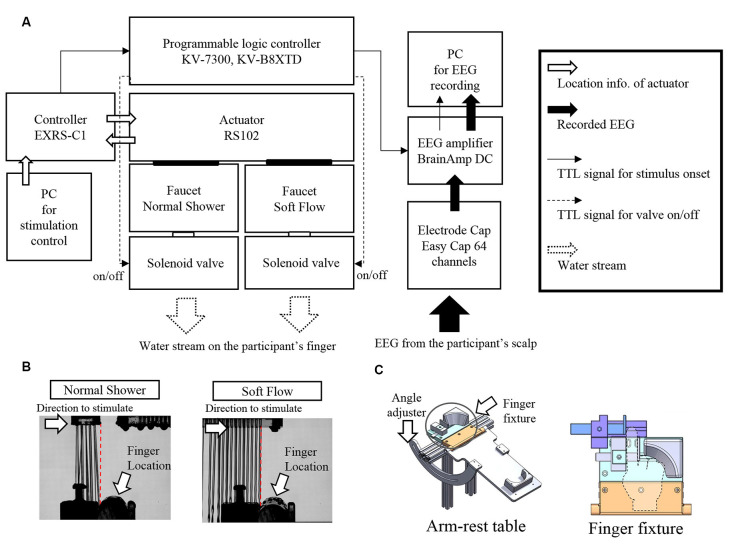
Stimulation device and water stream used in the electroencephalography (EEG) experiment. **(A)** Schematic representation of the stimulation device. **(B)** Pictures of touching a water stream with an acrylic rod (substitute for a finger) captured by a high-speed camera. **(C)** Schematic illustrations of arm and finger fixtures.

A stimulation device using a water stream was developed for this experiment (Figure 1A). Two faucets with different shapes were attached to an actuator (MISUMI, RS102, Tokyo, Japan). One faucet produced a normal shower water stream, whereas the other produced soft flow of water. The water stream from the latter faucet was straighter and induced softer touch sense compared to that of the normal shower (details in subsequent section). The device could randomly switch between the two types of faucets for water stimulation during experimentation for each trial, which enabled us to conduct a discrimination task, typically termed the “oddball task,” using water stream as a stimulus. Water streams were continuously delivered to the faucets in parallel. Solenoid valves (TOTO Limited, THE13, Fukuoka, Japan) attached to the faucet controlled whether the water stream was released or stopped. A controller (MISUMI, EXRS-C1, Tokyo, Japan) of the actuator monitored the position of the faucet. When the actuator moved the faucet to the programmed position, transistor–transistor logic signal was sent to the EEG amplifier as a trigger signal of touch onset using a programmable logic controller (KEYENCE, CPU: KV-7300, KV-B8XTD, Osaka, Japan). The aforementioned system was attached to a stage made of aluminum frames. A basin with a drain hose was attached to the stage and covered by a thin plastic wall preventing water splashes and visualization of the water stream.

#### Water Stream Stimuli

Two types of water stream stimuli (normal shower and soft flow shower) were used in the EEG experiment. Before the stimulation, the water stream was released by controlling a solenoid valve. The actuator position was placed at a distance from the finger such that the water stream or any water splash would not contact the finger. The actuator was controlled to move from the right side to the participant’s left hand. When the actuator reached the intended position where the water stream first touched the right end of the left index finger, the transistor–transistor logic signal as stimulus onset trigger was sent to the EEG amplifier. This onset trigger timing was confirmed by a pilot experiment using an acrylic rod instead of a finger. The position of the actuator, where the water stream first touched the right end of the acrylic rod, was searched in 0.1-mm steps using a high-speed camera (Photron, FASTCAM Mini UX100, Tokyo, Japan; Figure 1B). The discrepancy between timing of the trigger and that of water touching was kept below 1 ms (0.1 mm).

### Experimental Procedures

Participants were fitted with an EEG electrode cap and received instructions about the task. In the experimental room, participants sat on a comfortable chair and placed their arm and hand on the arm-rest table (Figure 1C) of the stimulation device in a palm-up posture. The angle of the table could be adjusted seamlessly for each participant to feel comfortable. To constrain incidental movement of the participants’ finger, participants were required to place the index finger of their left hand into a finger fixture. For adjustment of the program to control the actuator based on individual finger shape, we simulated the finger touch position using a scale. Trigger timing was adjusted by the finger touch position.

Before the beginning of experimentation, participants wore canal-type earphones (ZERO AUDIO, DX211-WB, Kyoto, Japan) to hear white noise as a masking sound. Participants heard the sound induced by movement of the actuator without touching the water stream. Volume level of the white noise was adjusted such that participants could not hear device-induced noise. The maximum volume was 70 dB for adjustments.

Participants were instructed to view the center of a box which occluded sight of the water stream in front of their face and to maintain their posture. Participants were encouraged to interfere with movement of the index finger of the left hand. Participants were required to count the touches of the water streams during the experimental block as target stimuli. The order of targets was counterbalanced across all participants. At the beginning of the experimental block, after a 10-s blank period, one of the water streams touched the index finger’s inner surface of the left hand.

Touching continued for 2,000 ms. After the stimulation offset, a resting period was inserted. The interstimulus interval was varied from 5,450 to 5,700 ms by 50-ms steps. The average interstimulus interval was 5,575 ms. In one block, participants touched the water stream 60 times. The standard stimulus was presented 48 times, whereas the target stimulus was presented 12 times. After all trials in one block, participants were required to report the number of target stimuli based on their internal count. The next block started with a short break. Each session comprised five blocks without changing the target stimulus. Breaks were provided as per participants’ request. The second session started with the altered target stimulus. Participants perceived the water stream 600 times in total, divided into 10 blocks.

### EEG Recordings

EEG waveforms were recorded using the BrainAmp DC amplifier (Brainproducts, GmbH, Munich, Germany). The sampling rate was 1,000 Hz. The reference electrode was placed on the nose tip, and the grand electrode for the scalp EEG was placed on the back of the neck. The 64-channel active electrodes were distributed over the whole scalp according to the 10-10 international standard position using an EEG recording cap (EasyCap, GmbH, Herrsching, Germany). Horizontal electrooculograms (EOGs) were recorded by bipolar surface electrodes placed on the left and right outer canthus. The vertical EOG was recorded by bipolar surface electrodes placed above and under the left eye (eyebrow). The hardware filter settings were as follows: the low frequency cutoff was 0.016 Hz (time constant, 10 s), whereas the high frequency cutoff was 1,000 Hz. The impedance at each electrode was maintained at least below 50 kΩ and typically below 10 kΩ. The grand average of impedance across all participants and electrodes at the beginning of recording was 6.24 kΩ (*SD* = 1.85).

### EEG Data Analysis

EEG waveforms were analyzed using EEGLAB eeglab14_1_1b (Delorme and Makeig, 2004) under the Matlab R2018a (MathWorks, Natick, MA, USA). First, the recorded waveforms were digitally filtered by 1 Hz high-pass and 40 Hz low-pass filters. Continuous EEG waveforms were segmented from −600 to 1,200 ms after stimulus touch timing. Independent component analysis (ICA) decomposition with the Infomax method was applied to the segmented data. Based on the component waveforms, artifact-contaminated trials were discarded according to maximum and minimum amplitude, mean trial probability, kurtosis value, and spectrum power. Details are described in the Supplementary Material. Another ICA was applied to trial-rejected datasets, and the component waveforms were obtained anew. Dipole locations for all components were estimated using dipfit2 (EEGLAB plug-in using FieldTrip toolbox functions; Oostenveld et al., 2011).

For event-related potential (ERP) analysis to differentiate P300 deflection by standard and target stimulus presentation, we conducted cluster-based IC rejection to discard the eye-movement related EEG deflection. After this rejection, we selected the Pz electrode and calculated ERP waveforms. For statistical analysis, the max value during 300–800 ms across all trials was used for P300 amplitude for each participant. Wilcoxon-signed rank test was conducted, and the *p* values were corrected using the Bonferroni method.

Regardless of target/standard stimulus, we visualized brain activation related to the difference between normal and soft water streams, and calculated alpha power. For further analyses, we modified the previously mentioned data set by merging all trials into one condition of water stream shape regardless of presentation probability (target/standard). For alpha asymmetry, we calculated the values for the stimulated period (2 s) using the traditional formula [ln(F4) − ln(F3) (Allen et al., 2004)] and statistically tested the difference between the two streams using Wilcoxon-signed rank test with Bonferroni correction of the *p*-value. For alpha suppression, we calculated event-related spectrum power (ERSP). Using the merged dataset, we conducted ICA clustering analysis and derived 10 clusters. Based on the hypothesis of alpha wave distribution, we focused on the parieto-occipital cluster. ERSPs at the cluster were calculated for −100 to 800 ms and 3–30 Hz. The baseline was a period between −300 and −50 ms. The difference of ERSPs between two water streams was statistically tested using Monte Carlo permutation statistics with cluster correction (channel neighbor parameters: triangulation, clustering method: max-sum) implemented in FieldTrip toolbox (Oostenveld et al., 2011).

### Follow-Up Water Flow Evaluation Task

All participants were recruited for a follow-up evaluation task more than 6 months after participation in the EEG experiment. Participants sat on a comfortable chair in a light room and adopted an identical posture to that in the EEG experiment. Participants received identical water streams on the index finger of their left hand. One of the two types of water streams was alternately delivered to the participant’s finger. The order of stimuli was counterbalanced across participants. Participants were required to rate the water streams after each stimulation in terms of “richness” and “comfort,” as conducted in the EEG experiment. In total, 10 stimulations and ratings were conducted. The averaged score across all ratings was used as an evaluation of each participant.

## Results

### P300 to the Water Stream Stimulus

**Figure 2:**
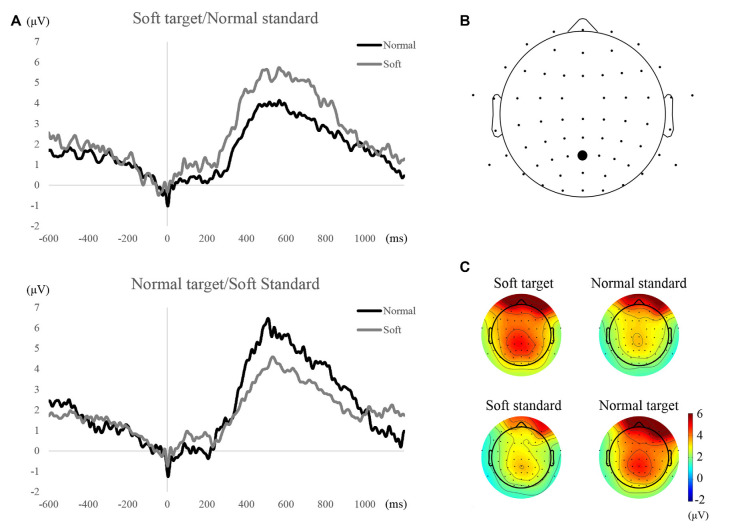
Waveforms and topographical maps of P300 components. **(A)** Grand averaged event-related potential (ERP) waveforms at the Pz electrode for each condition and stimulation. **(B)** The location of Pz electrode. **(C)** Scalp distribution at amplitudes averaged across 300–800 ms time period.

The grand averaged ERP waveforms at Pz electrode for all conditions are plotted in Figure 2. Based on visual inspection of the waveforms, ERP waveforms to the target stimulus were greater than those to standard stimuli regardless of the shape of the water stream. The time period in which the waveforms to target stimuli were greater than those to standard stimuli was from about 300–800 ms. This time period is typical for P300 to low-frequency target stimuli. Statistical analysis of the maximum values during that time period revealed significant differences between target and standard stimuli for both blocks (soft target/normal standard condition, *Z* = 2.62, *p* < 0.05, *r* = 0.34, normal target/soft standard, *Z* = 3.92, *p* < 0.01, *r* = 0.51).

### Subjective Rating for Each Shower Shape

**Figure 3:**
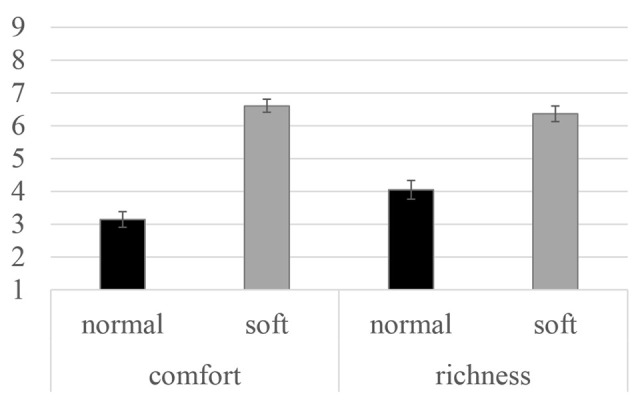
Subjective rating scores for water stream perception averaged across 23 participants. Error bars indicate the standard errors for each condition.

Participants evaluated the richness and comfort of perceived water streams by normal and silky shower. The averaged values of subjective ratings in the follow-up evaluation of water streams are illustrated in Figure 3. Both subjective rating scores were higher when participants received the soft water stream than when they received the normal shower. The difference in scores between two types of water streams were statistically analyzed using a *t*-test with corrected *p*-values (Bonferroni method *p*-value correction). Significant differences in both ratings were observed (*t*_(29)_ = 6.01, *p* < 0.01, Cohen’s *d* = 1.09 for comfort; *t*_(29)_ = 11.05, *p* < 0.01, Cohen’s *d* = 2.02 for richness).

### Alpha Asymmetry for Each Shower Shape

**Figure 4:**
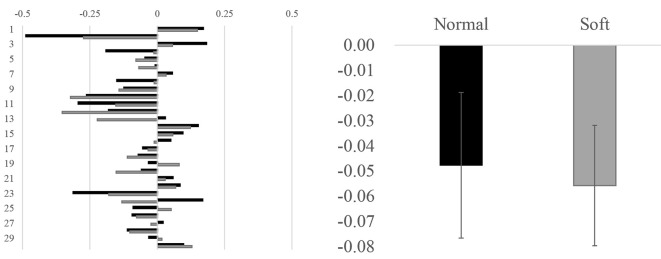
Alpha asymmetries in individuals for each water stream and averaged value for each stimulus. In the left panel, alpha asymmetry in each individual is illustrated. The *y*-axis denotes participant numbers, whereas the *x*-axis denotes the value of alpha asymmetries (microV^2^). In the right panel, the alpha asymmetries are averaged across all participants. The *y*-axis denotes the value of alpha asymmetries (microV^2^).

No significant difference between different water streams was observed in alpha asymmetry index. The alpha asymmetry for each water stream was almost identical (−0.05 for normal shower vs. −0.06 for soft flow, *Z* = 0.61, n.s.; Figure 4, right panel). Almost one-third of the participants showed the opposite (positive) value to the trend (negative value), which suggests great interindividual variability in alpha asymmetries (Figure 4, left panel).

### Alpha Suppression for Each Shower Shape

**Figure 5:**
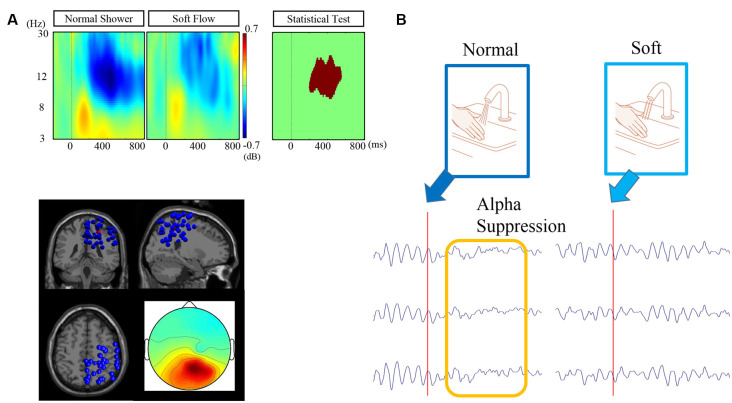
**(A)** Event-related spectrum power (ERSP) for each condition, dipole locations, and scalp topography of the target cluster. **(B)** Schematic representation of differentiated alpha suppression by touch with normal and silky water streams. The illustrated waveforms are typical waveforms at the occipital electrodes before and after stimulation with normal and silky water streams.

We observed alpha suppression at the parieto-occipital cluster after touching a water stream (Figure 5). The centroid of the parietal cluster was located at *X*, 26; *Y*, −41; *Z*, 49. More than half of the ICs’ dipole positions (41/76) are in the parietal area, including postcentral, superior parietal, precuneus, and angular gyrus (Table 2). Statistical analysis revealed that alpha suppression to the normal shower was significantly greater than that to the silky shower stimulus. The significant time frequency window was 8–17 Hz and 200–600 ms.

**Table 2:** Dipole characteristics for each independent component involved in target cluster 3.

AAL name of the dipole position	*N* of ICs	Average RV	SD of RV
Postcentral_R	14	10.12	4.19
Precuneus_R	12	7.63	3.03
Parietal_Sup_R	9	10.15	4.55
Angular_R	6	8.97	4.18
Precentral_R	5	13.03	2.23
Parietal_Inf_R	5	5.41	2.41
Cingulum_Mid_R	4	7.82	3.99
Cingulum_Post_R	3	6.64	4.36
Precuneus_L	3	9.43	2.15
Cingulum_Mid_L	2	7.02	0.24
Cingulum_Post_L	2	3.69	0.87
Paracentral_Lobule_R	2	4.93	1.17
Occipital_Sup_R	2	5.54	0.49
Frontal_Sup_R	2	13.81	2.47
Insula_R	1	9.71	0
Temporal_Mid_R	1	6.92	0
Paracentral_Lobule_L	1	3.22	0
Frontal_Mid_R	1	10.85	0
Occipital_Mid_R	1	2.92	0

*For example, at the top of the rows, the dipole positions of 14 independent components (ICs) fall in the area named “Postcentral_R” (the right postcentral gyrus) based on the automated anatomical labeling (AAL) atlas 3. The average residual variance of dipoles across all ICs of “Postcentral_R” was 10.12, and the standard deviation was 4.19. AAL, automated anatomical labeling; IC, independent component; RV, residual variance*.

### Correlation Analyses of Subjective Ratings and EEG Component

**Figure 6:**
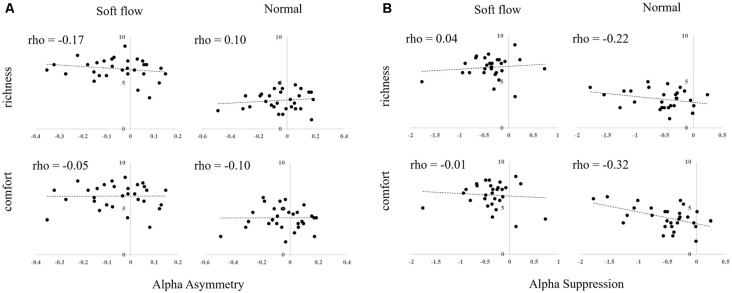
Scatter plots of subjective rating scores and EEG responses to water streams. **(A)** Alpha asymmetry. **(B)** Alpha suppression.

We calculated the Spearman rho correlation coefficient to analyze the relationship between EEG activity (alpha asymmetry and alpha suppression) and subjective rating scores (comfort and richness) obtained by follow-up experiments. No significant correlations were observed. Scatter plots are illustrated in Figure 6.

## Discussion

In this study, we aimed to test two hypotheses: first, whether a water stream as a tactile stimulus could induce P300 and whether amplitudes were different between target and standard stimulation in the typical oddball task; second, whether tactile comfort induced by a water stream could be indexed by alpha oscillation. Significant modulation of P300 was observed in this experiment, suggesting that the methodology used in this experiment was effective for electrophysiological studies using water streams. Alpha suppression was significantly modulated by the shape of the water stream, which differentiated subjective reports of comfort and richness, implying that alpha oscillation could be involved in affective processing when touching water streams.

P300 amplitude was greater for the target stimulus regardless of the shape of the water stream. This suggested that P300 could be elicited by the shape of the water stream in a similar manner as that of typical visual and/or tactile oddballs using surface toughness. Scalp topography was also very typical of P300 component during the oddball task. In the topographical map of P300, positive deflections were also observed over the frontal area. This is possibly related to eye movement-related noise deflections, as we did not instruct participants to refrain from eye blinking when touching the water stream. We tried to discard eye movement-related EEG waveforms using ICA. We successfully detected and excluded a cluster distributed over the frontal pole before ERP calculation. However, the frontal distributed positive deflection remained in the resulting EEG data, suggesting that there were difficulties in ICA-based removal of EOG-related activity from EEG waveforms recorded in free eye movement conditions. In these results, we observed clear boundaries between frontal and parietal positive deflections, which supported the conclusion that the P300 difference at Pz was not contaminated by EOG-related positive deflections.

Furthermore, we calculated the alpha asymmetry index for each EEG waveform to different water streams; however, there was no significant difference between them. Subjective reports have revealed that the comfort and richness evaluation for soft flow was significantly higher than that for normal shower, which led to the assumption that alpha asymmetry during perception of soft flow is greater than that during normal shower because negative emotional states induce greater alpha asymmetry than do normal and positive emotional stimuli. However, many studies using alpha asymmetry have adopted longer time periods for analysis. For example, the waveforms when viewing video clips ranging from 6 s to several tens of minutes were used for calculating spectrum powers (Davidson et al., 1999; Allen et al., 2001; Meyer et al., 2014). In this study, water stream stimulation continued for only 2 s, which may have been insufficient for alpha asymmetry analysis. In the future, experiments with longer stimulation periods are required to clarify whether alpha asymmetry can be an emotional index of water stream perception.

Finally, we assumed that alpha suppression at the parietal area could be modulated by different stimuli. Compared to normal shower, soft flow showed significantly lower alpha suppression of a cluster at the parietal area. The relationship between emotional response and alpha suppression has previously been reported (Kostyunina and Kulikov, 1996; Swingle, 2013), which suggests that alpha suppression could be related to emotional responses to water stream stimulation. The cluster involved many ICs with dipole locations in the postcentral, superior parietal, precuneus, and angular gyrus, which suggested that the primary and secondary somatosensory area and the sensory association area could be related to the water pleasantness perception. However, because the correlations between alpha suppression and subjective evaluations were not significant, we were unable to conclude that alpha suppression was directly related to the feeling of comfort or richness when perceiving a water stream. In addition, we have to note that nonparametric statistical tests were used as we could not presume a normal distribution; therefore, we could only determine that the rank (high or low amplitude) was consistent across participants. Previous studies have demonstrated that alpha suppression was greater when participants paid attention to the stimulus (Siegel et al., 2008; Wyart and Tallon-Baudry, 2008), especially using visuospatial attention. In this study, exogenous covert attention is a candidate for modulation of alpha suppression after touching a water stream. Future research is required to clarify the relationships among the amount of induced attention, alpha suppression, and tactile comfort.

To conclude, we have demonstrated that water stream could be used as tactile stimulation for electrophysiological experiments. During a typical oddball task, water stream-induced P300 components and a difference between target and standard stimuli were observed. Furthermore, we demonstrated that less alpha suppression was related to comfort/richness of a water stream, although no direct correlation between them was observed. Future research is required to clarify the relationships among the amount of induced attention, alpha suppression, and tactile comfort. Our findings may be used as an electrophysiological index of affective evaluation for engineering.

## Data Availability Statement

The datasets generated for this study will not be made publicly available. As a rule of the Ethical Committee, we cannot disclose the raw biological data acquired from human participants.

## Ethics Statement

The studies involving human participants were reviewed and approved by TOTO Limited, Research Institute, Ethical Committee. Written informed consent for participation was not required for this study in accordance with the national legislation and the institutional requirements.

## Author Contributions

NK, SM, RY, TO and SY have read, discussed and approved the manuscript for submission. NK, SM, RY, TO and SY discussed to design the behavioral and EEG experiment. NK, SM, RY and TO designed and developed the device. NK, SM and RY collected the data. NK wrote the article.

## Conflict of Interest

SM, RY, and TO were employed by the company TOTO Limited. The remaining authors declare that the research was conducted in the absence of any commercial or financial relationships that could be construed as a potential conflict of interest.
