# Corrigendum: The Shape of Water Stream Induces Differences in P300 and Alpha Oscillation

**DOI:** 10.3389/fnhum.2020.587733

**Published:** 2020-10-21

**Authors:** Noriaki Kanayama, Shumpei Mio, Ryohei Yaita, Takahiro Ohashi, Shigeto Yamawaki

**Affiliations:** ^1^Human Informatics Research Institute, National Institute of Advanced Industrial Science and Technology (AIST), Tsukuba, Japan; ^2^Center for Brain, Mind and KANSEI Sciences Research, Hiroshima University, Hiroshima, Japan; ^3^TOTO Limited, Research Institute, Chigasaki, Japan

**Keywords:** water, EEG, P300-event related potential, alpha oscillations, touch

In the original article, there was a mistake in Figure 6 as published. **In the figure, the locations of the scatter plot were wrong. In original Figure 6B, a plot for Softflow x comfort (left bottom) was a plot for Normal x richness (right up)**. The corrected Figure 6 appears below.

**Figure 6:**
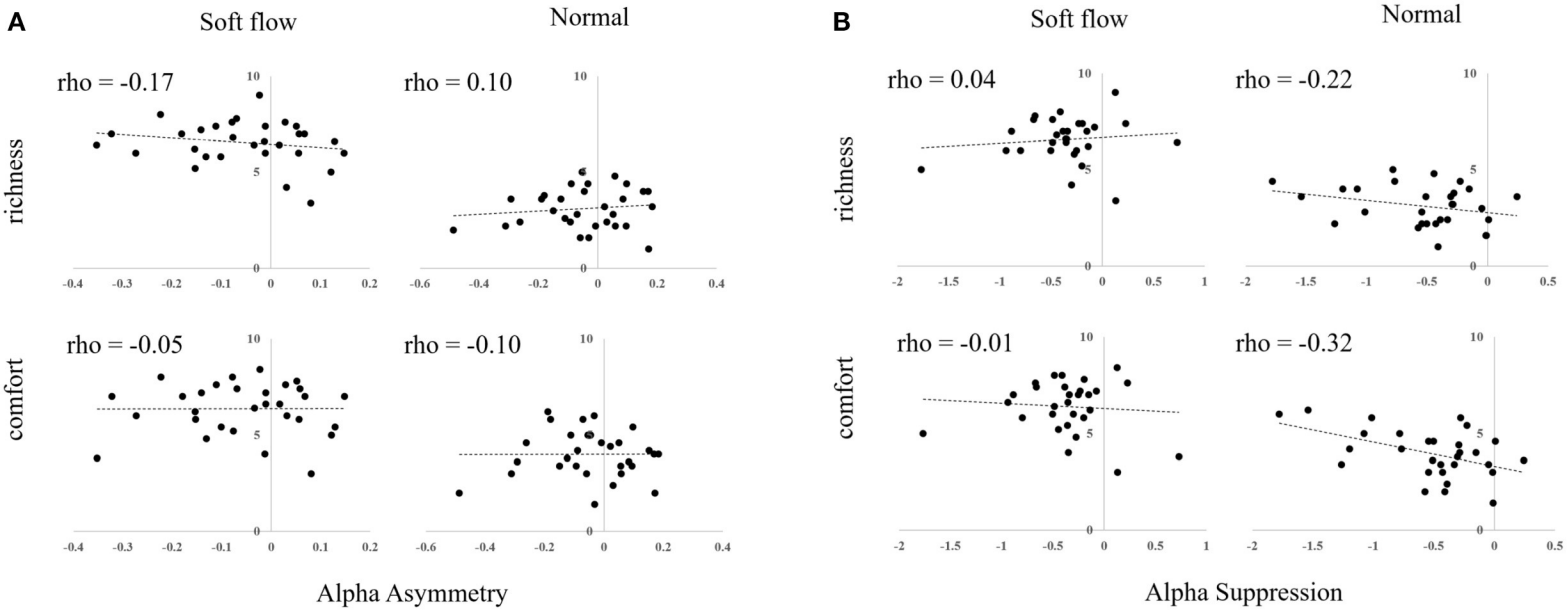
Scatter plots of subjective rating scores and EEG responses to water streams. **(A)** Alpha asymmetry. **(B)** Alpha suppression.

The authors apologize for this error and state that this does not change the scientific conclusions of the article in any way. The original article has been updated.

