# Supplementary material for: The Shape of Water Stream Induces Differences in P300 and Alpha Oscillation
Source: Front Hum Neurosci. 2020 Jan 20;13:460. doi: 10.3389/fnhum.2019.00460 (PMC6984336; doi:10.3389/fnhum.2019.00460)
Supplement: Supplementary file 1 [file Data_Sheet_1.pdf]

Supplementary Material

EEG Data Analysis

EEG waveforms were analyzed using EEGLAB eeglab14_1_1b (Delorme & Makeig, 2004) under the Matlab R2018a (MathWorks, Natick, MA, USA). First, the recorded waveforms were digitally filtered. High-pass (finite impulse response, passband edge = 1 Hz, transition band width = 1 Hz, cut-off frequency = 0.5 Hz) and low-pass filters (finite impulse response, passband edge = 40 Hz, transition band width = 10 Hz, cut-off frequency = 45 Hz) were applied to the raw data. Continuous EEG waveforms were segmented based on the trigger timing, when the water stream stimulus touched a participant's index finger of the left hand. The segment started from -600 ms and continued to 1,200 ms after stimulus touch timing. Independent component analysis (ICA) decomposition with the Infomax method was applied to the segmented data. The 64 independent components were obtained for each participant. Based on the component waveforms, artifact-contaminated trials were discarded according to the following criteria: (1) simple threshold of amplitude ± 300, 350, or 400 μV for the electrodes except around the eyes (Fpz, Fp1, Fp2, AF3, AF4, AF7, AF8) as too many trials would be discarded if electrodes near the eyes were included; (2) threshold using 3 SDs of the mean

trial probability distribution for each electrode; (3) threshold using 5 SDs of kurtosis value of the waveforms in one trial; (4) threshold using spectrum power between 3 and 30 Hz for each electrode. Trials showing less than -100 dB or typically more than 50 dB (or 60 dB for some participants) were discarded. Criteria were modified across participants to maintain the rejection rate under 10%. The trials were also rejected by visual inspection before automatic rejection using the above-mentioned criteria. The consistency between these methods were higher than 90%. For reproducibility of data, we adopted the automatic rejection.

Another ICA was applied to trial-rejected datasets, and the component waveforms were obtained anew. Dipole locations for all components were estimated using dipfit2 (EEGLAB plug-in using FieldTrip toolbox functions; Oostenveld, Fries, Maris, & Schoffelen, 2011). A head model was created using the Montreal Neurological Institute standard coordinate system, and the typical electrode locations for 10-10 positions were allocated to each electrode.

For event-related potential analysis to differentiate P300 deflection by standard and target stimulus presentation, we conducted cluster-based IC rejection. First, based on the dipole location and scalp topography, all ICs were separated into 10 clusters. From these clusters, a cluster, of which the scalp distribution was biased to the frontal pole, was considered as

blinking and eye movement-induced artifact. The IC was discarded from the data, and all remaining (theoretically intact from eye movement noise) components were back-projected to the 64 electrodes. As a typical electrode for P300, we selected the Pz electrode and calculated ERP waveforms. For statistical analysis, the max value during 300-800 ms across all trials was used for P300 amplitude for each participant. Wilcoxon signed rank test was conducted, and the p-values were corrected using the Bonferroni method.

Regardless of target/standard stimulus, we visualized brain activation related to the difference between normal and soft water streams and calculated alpha power deflections. For further analyses, we modified the previously mentioned data set by merging all trials into one condition of water stream shape regardless of presentation probability (target/standard). For alpha asymmetry, we calculated the values for the stimulated period (2 second) using the traditional formula, (ln(F4) – ln(F3)), (Allen, Coan, & Nazarian, 2004) and statistically tested the difference between the two streams using Wilcoxon signed rank test with Bonferroni correction of the p-value. For alpha suppression, we calculated event-related spectrum power (ERSP). Using the merged dataset, we conducted another ICA and dipole estimation for each IC and calculated ERSP of each component. Based on the dipole location, scalp topography, and spectrum power, all ICs

were separated into 10 clusters. Based on the hypothesis of alpha wave distribution, we focused on the parieto-occipital cluster. ERSPs at the cluster were calculated for -100 to 800 ms (separated into 200 time bins) and 3-30 Hz (separated in 50 frequency bins) using wavelet cycles 2 at the lowest frequency and 4 at the highest frequency. The baseline was a period between -300 ms and -50 ms. The difference of ERSPs between two water streams was statistically tested using Monte Carlo permutation statistics with cluster correction (channel neighbor parameters: triangulation, clustering method: max-sum) implemented in FieldTrip toolbox (Oostenveld, Fries, Maris, & Schoffelen, 2011).
